# Distribution of Exonic Variants in Glycogen Synthesis and Catabolism Genes in Late Onset Pompe Disease (LOPD)

**DOI:** 10.3390/cimb45040186

**Published:** 2023-04-01

**Authors:** Paola De Filippi, Edoardo Errichiello, Antonio Toscano, Tiziana Mongini, Maurizio Moggio, Sabrina Ravaglia, Massimiliano Filosto, Serenella Servidei, Olimpia Musumeci, Fabio Giannini, Alberto Piperno, Gabriele Siciliano, Giulia Ricci, Antonio Di Muzio, Miriam Rigoldi, Paola Tonin, Michele Giovanni Croce, Elena Pegoraro, Luisa Politano, Lorenzo Maggi, Roberta Telese, Alberto Lerario, Cristina Sancricca, Liliana Vercelli, Claudio Semplicini, Barbara Pasanisi, Bruno Bembi, Andrea Dardis, Ilaria Palmieri, Cristina Cereda, Enza Maria Valente, Cesare Danesino

**Affiliations:** 1 IRCCS Mondino Foundation, 27100 Pavia, Italy; 2 Department of Molecular Medicine, University of Pavia, 27100 Pavia, Italy; 3 ERN-NMD Center of Messina for Neuromuscular Disorders, Department of Clinical and Experimental Medicine, University of Messina, 98125 Messina, Italy; 4 Neuromuscular Unit, Department of Neuroscience RLM, University of Torino, 10126 Torino, Italy; 5 Neuromuscular and Rare Diseases Unit, BioBank of Skeletal Muscle, Peripheral Nerve, DNA and Dino Ferrari Center, IRCCS Foundation Ca’ Granda Ospedale Maggiore Policlinico, 20100 Milan, Italy; 6 Department of Clinical and Experimental Sciences, NeMO-Brescia Clinical Center for Neuromuscular Diseases, University of Brescia, 25121 Brescia, Italy; 7 Department of Neuroscience, Catholic University, 00100 Rome, Italy; 8 Department of Clinical and Experimental Medicine, University of Messina, 98125 Messina, Italy; 9 Department of Medical, Surgical and Neurological Sciences, University of Siena, “Le Scotte” Hospital, 53100 Siena, Italy; 10 Fondazione IRCCS San Gerardo, Centro Ricerca Testamenti, Monza-European Reference Network–MetabERN, 20900 Monza, Italy; 11 Department of Clinical and Experimental Medicine, Neurological Clinics, University of Pisa, 56100 Pisa, Italy; 12 Centre for Neuromuscular Disease, CeSI, University “G. d’Annunzio”, 66100 Chieti, Italy; 13 Dipartimento di Ricerca Malattie Rare, Istituto Mario Negri IRCCS, 24020 Ranica, Italy; 14 Department of Neurosciences, Biomedicine and Movement Sciences, Section of Clinical Neurology, University of Verona, 37100 Verona, Italy; 15 Department of Neurosciences, University of Padova, 35100 Padova, Italy; 16 Cardiomiologia e Genetica Medica, Dipartimento di Medicina Sperimentale, Seconda Università di Napoli, 80100 Napoli, Italy; 17 Neuroimmunology and Neuromuscular Diseases Unit, Fondazione IRCCS Istituto Neurologico Carlo Besta, 20100 Milano, Italy; 18 Regional Coordinator Centre for Rare Diseases, University Hospital “Santa Maria della Misericordia”, 33100 Udine, Italy; 19 Center of Functional Genomic and Rare Diseases-Buzzi Children’s Hospital, 20100 Milano, Italy

**Keywords:** late-onset Pompe disease (LOPD), exonic variants, glycogen synthesis, glycogen catabolism, genotype–phenotype correlates, genetic modifiers

## Abstract

Pompe disease (PD) is a monogenic autosomal recessive disorder caused by biallelic pathogenic variants of the *GAA* gene encoding lysosomal alpha-glucosidase; its loss causes glycogen storage in lysosomes, mainly in the muscular tissue. The genotype–phenotype correlation has been extensively discussed, and caution is recommended when interpreting the clinical significance of any mutation in a single patient. As there is no evidence that environmental factors can modulate the phenotype, the observed clinical variability in PD suggests that genetic variants other than pathogenic GAA mutations influence the mechanisms of muscle damage/repair and the overall clinical picture. Genes encoding proteins involved in glycogen synthesis and catabolism may represent excellent candidates as phenotypic modifiers of PD. The genes analyzed for glycogen synthesis included *UGP2,* glycogenin (*GYG1*-muscle, *GYG2*, and other tissues), glycogen synthase (*GYS1*-muscle and *GYS2*-liver), *GBE1*, *EPM2A, NHLRC1*, *GSK3A,* and *GSK3B.* The only enzyme involved in glycogen catabolism in lysosomes is α-glucosidase, which is encoded by *GAA*, while two cytoplasmic enzymes, phosphorylase (*PYGB*-brain, *PGL*-liver, and *PYGM*-muscle) and glycogen debranching (*AGL*) are needed to obtain glucose 1-phosphate or free glucose. Here, we report the potentially relevant variants in genes related to glycogen synthesis and catabolism, identified by whole exome sequencing in a group of 30 patients with late-onset Pompe disease (LOPD). In our exploratory analysis, we observed a reduced number of variants in the genes expressed in muscles versus the genes expressed in other tissues, but we did not find a single variant that strongly affected the phenotype. From our work, it also appears that the current clinical scores used in LOPD do not describe muscle impairment with enough qualitative/quantitative details to correlate it with genes that, even with a slightly reduced function due to genetic variants, impact the phenotype.

## 1. Introduction

Pompe disease (PD) is a monogenic autosomal recessive disorder caused by biallelic pathogenic variants of the GAA gene encoding lysosomal alpha-glucosidase; its loss causes glycogen storage in lysosomes, mainly in the muscular tissue [1]. Late-onset PD (LOPD) phenotypes, including childhood, juvenile, and adult-onset forms, are characterized by proximal muscle weakness and respiratory insufficiency without cardiac involvement. Despite these common features, wide clinical variability is observed regarding the onset age, rate of disease progression, and clinical pattern, including the relative involvement of motor vs. respiratory muscles [2].

To date, over 300 pathogenic mutations in the *GAA* gene have been identified. According to their impact on protein function, they have been classified as “very severe”, “potentially less severe”, “potentially mild”, and “less severe” [3], with an attempt to correlate them to the overall clinical severity [4,5]. In patients with LOPD, various combinations of pathogenic *GAA* alleles result in residual enzyme activity ranging from about 2 to 30% of normal values. However, the observed clinical variability is only partially explained by the residual enzyme activity, and the age of onset and the severity of disease progression are not easily predictable [6].

The existence of a stricter genotype–phenotype correlation, suggested by the phenotypes observed in some patients carrying homozygous variants, has been discussed in adults by Musumeci et al. (2015) [7] for the common mutation c.-32-13T>G, and in the case of a neonatal presentation of Pompe carrying the mutation c. 2560C>T (p.Arg854X), it has been discussed by Martinez et al. (2017) [8]. However, the same missense and splicing mutations, resulting in complete or partial absence of GAA activity, can be found in both infantile PD and LOPD in association with a second severe or milder mutations, respectively [9,10,11,12].

These observations, in keeping with the wide intrafamilial variability observed by us and others, recommend caution in interpreting the clinical significance of any mutation in a single patient [13,14].

As there is no evidence that environmental factors can modulate the disease phenotype, the observed clinical variability of PD (with a poor genotype–phenotype correlation), as well as the unpredictable response to enzyme replacement therapy (ERT), may indicate that genetic variants other than pathogenic GAA mutations may influence the mechanisms of muscle damage/repair and the overall clinical picture [15].

For instance, Bergsma et al. (2019) [16] provided evidence that the synonymous variant c.510C>T was uniquely present on the IVS1 allele in patients with childhood onset but absent in patients with late onset. Indeed, skeletal muscle is a highly adaptable tissue that responds to environmental and physiological challenges through changes in size, fiber type, and metabolism, which are under the control of many different genes; for instance, the autophagy pathway has been implicated in the pathogenesis of PD (Raben, 2012) [17].

Genes encoding proteins involved in the synthesis and catabolism of glycogen (reviewed in Adeva-Andany et al., 2016) [18] may represent excellent candidates as phenotypic modifiers of PD. Glycogen synthesis is a complex process in which glucose molecules are available as UDP-glucose and are synthetized by *UGP2* and glycogenin (*GYG1*-active in muscle, and *GYG2* in other tissues), which initiate synthesis by autoglycolysation. Glycogen synthase, encoded by *GYS1* in the muscle and *GYS2* in the liver, adds glycosil moieties to the growing short chain by an α-1,4-glycosidic linkage, and the last step is accomplished by the branching enzyme (*GBE1*), which adds α-1,6-glycosidic bonds at regular intervals. Laforin and Malin (encoded by *EPM2A* and *NHLRC1*, respectively) may also influence glycogen assembly, even if their exact functions are still unclear.

GSK3A and GSK3B are multifunctional proteins implicated in the control of *GYS1*, and they are both more highly expressed in the muscle than in the liver. As for glycogen catabolism, glucose molecules can be obtained from glycogen in both lysosomes and the cytoplasm. In lysosomes, the only enzyme involved is α-glucosidase, coded by *GAA*, while two cytoplasmic enzymes, glycogen phosphorylase (*PYGB,* in the brain; *PYGL*, in the liver; and *PYGM*, in the muscle) and glycogen debranching (*AGL*) are needed to obtain glucose 1-phosphate or free glucose. 

Here, we report the potentially relevant variants in genes related to glycogen synthesis and catabolism, which were identified by whole exome sequencing in a group of 30 patients with LOPD. Our hypothesis was that a combination of such variants, each of which contributes to a small additional effect, may modulate the overall clinical picture. 

## 2. Patients and Methods

### 2.1. Patients

Patients diagnosed with LOPD were recruited and clinically characterized at the participating centers. Informed consent was obtained from all patients. The study was approved on 26 September 2016 by the Ethical Committee (EC) of the University of Pavia, and subsequently by the ECs of the hospitals caring for the patients included in the study.

Calculation of 6 min walking test (6 mwt) as a percentage of the expected normal value for sex, age, and body mass index was performed as reported by Enright et al. (2003) [19]. Genetic diagnosis was performed in most cases at the Pavia Center. All patients shared the same genotype at allele 1 [c.-32-13T>G], whereas a limited number of common Italian pathogenic variants were found in allele 2 (Table 1). 

### 2.2. Whole Exome Sequencing 

Whole exome sequencing libraries were prepared from genomic DNA (1.5 µg extracted from peripheral whole blood) using a commercial target enrichment kit (Agilent SureSelectXT HumanAllExon V5 + UTRs; Agilent Technologies, Santa Clara, CA, USA) and sequenced on a HiSeq1000 platform (paired-end 2 × 100 nt; Illumina, San Diego, CA, USA).

Briefly, reads were quality-filtered and aligned to the reference human genome sequence (GRCh37/hg19) with an ISAAC aligner (Raczy, C. et al. Isaac: ultra-fast whole-genome secondary analysis on Illumina sequencing platforms. Bioinformatics 29, 2041–3, 2013). Genomic variant annotation was carried out with VarSeq v1.4.5 (Golden Helix, Inc., Bozeman, MT, USA), and only variants with a minimum quality score of 20 and a minimum read depth of 30X were included in the downstream analysis.

Variants reported in gnomAD with a population frequency of >5% were excluded. We further prioritized the candidate variants using additional bioinformatics tools, such as PredictSNP, Mutation Assessor, VEP, SNPs&Go, PANTHER, PROVEAN, SNAP2, MAPP, and MutPred, and according to amino acid conservation scores. Finally, manual inspection of the Bam files, using Integrative Genomics Viewer (IGV), enabled the evaluation of the coverage and ensured the quality of the aligned reads. For the variants analyzed, the effect on protein function (damaging or tolerated) was reported by SIFT and polyphen. 

### 2.3. Statistical Analysis 

Differences in incidence of variants in the general population and in the sample analyzed were compared by Fisher’s exact test for count data due to the small sample size of observations for many of the alleles analyzed; differences were considered to be significant after Bonferroni correction, with *p*-value equal or below 0.0013 ⋍ 0.05/37.

## 3. Results and Discussion

Table 1 summarizes the demographic, genetic, and clinical data of patients, including the results for Walton score (ws), % 6 min walking test (6 mwt), % forced vital capacity (FVC), and the need for walking or respiratory support for each patient. The requirements for walking support (w+ or w-) and respiratory support (r+ or r-) were randomly distributed.

Table 2 shows the results (mean, SD) of ws, 6 mwt, FVC, and the number of cases requiring no, mild, or extensive walking and respiratory support for the whole group and for patients grouped according to the severity of the *GAA* pathogenic variant on the second allele [3,4].

Table 3a,b presents the number of expected vs. observed (e/o) variants and the Z score (a parameter reflecting the constraint or intolerance to variation) for synonymous (S) and non-synonymous (NS) variants identified in genes related to glycogen synthesis and catabolism (from gnomAD) and the number of cases with no, S, or NS variants. When the total number of variants exceeds 30, this implies that some cases carry more than one type of variant. 

Table 4a,b lists the exonic variants found in genes acting on glycogen synthesis and catabolism, and their incidence in the population (gnomAD) and in the samples studied. The codes of single cases carrying the variant or showing significant differences in an incidence versus the general population are in bold. 

Five genes (*UGP2, GYG2, GYS2, GBE1*, and *GSK3A*) presented variants with a significantly higher incidence in LOPD patients than in the general population. 

In Table 5a,b, we entered the z-score for clinical data (ws, 6 mwt, and FVC) for cases that were the only ones to carry a variant or carrying variants that had an incidence rate that was significantly different from the population.

## 4. Mutations and Variants

In the LOPD cohort studied here, patients carrying mutations defined as severe or less severe showed very similar results for all the clinical parameters tested (Table 2), at variances with the definition of severity. These data need to be taken with caution because of the small size of the sample; however, they highlight the need to collect detailed clinical data for a larger series of patients sharing the same genotype, as these comparisons may contribute useful data to better understand the natural history of the disease in well-defined subgroups of patients.

Similarly, when patients were grouped according to the second mutation, no differences were observed between the groups, confirming that the type of mutation alone was not enough to completely predict the severity of the clinical phenotype and, in detail, the predominant involvement of the motor or respiratory function (Table 2).

In some cases (Table 2, patients with the c.-32-13T>G/c.525delT genotype), the observation of worse results for ws was associated with better results for 6 mwt and FVC, which clearly indicates the need to discuss the different aspects of muscular and respiratory functions separately, using tests that are able to depict their components in detail. Indeed, ws is a parameter that gives only a rough global representation of the phenotype in a given moment of a patient’s history, without details on the clinical pattern/progression.

Mutation c.525delT (p.Glu176ArgfsTer45), reported in the gnomAD database with a frequency of 27/271120 is commonly found in infantile patients, but is not uncommon in Italian adult patients.^11^ In our database, including over 170 cases of LOPD, it was present in 24 cases. In addition, (our unpublished observation) in a group of 12 cases with genotype [c.-32-13T>G # c.525delT], for which detailed clinical data are available, we observed that disease-free life was below 40 years of age in eight cases, while clinical signs started much later in the remaining cases. Therefore, in late-onset adult patients carrying the mutation in the second allele (with the mutation c.-32-13T>G on the first), it is not uncommon to find a less severe clinical picture associated with the c.525delT mutation. In fact, in the present study (see Table 1 and Table 2), only case BGA02 showed a very early age of onset of the disease, while in the remaining cases, the age ranged between 25 and 65 years. Patient BGA02 shares the same SNPs with other cases bearing the same genotype, but lacks one synonymous variant in *GYG1* and has an additional non-synonymous variant in *GBE1* (rs2229519). The latter is defined as benign by polyphen (using straightforward physical and comparative considerations) but deleterious in SIFT (based on sequence homology and the physical properties of amino acids).

Overall, the worst results consistently found for ws, 6 mwt, and FVC were observed in patients carrying the c.1465G>A (p.Asp489Asn) on the second *GAA* allele, but the observation was limited to only two cases (ME01 and PT01); indeed, one of them also required the use of a walker. This mutation causes the GAA protein to remain as an inactive precursor without any residual enzyme activity, although it was found both in patients with an age of onset of less than 12 months and in patients with an age of onset of >12 years [20,21].

ME01 and PT01 did not differ from other cases in the presence of the number or types of variants observed (Table 4 and Table 5) and they were not among the cases that were carrying uncommon variants (Table 4 and Table 5).

Among the four cases with a very mild clinical presentation (ws = 0, 6 mwt = 100%, and FVC 100%) (Table 1 and Table 2), BR04, SM02, and SM04 share a very late age of onset, and the c.2237G>A mutation (classified as very severe) on the second allele, while ZA01 had a much earlier diagnosis (age 15), and carried the c.1927G>A mutation. In genes related to glycogen synthesis, only BR04 (Table 4a) carried a rare NS variant in GSK3B (benign according to polyphen and tolerated low confidence according to SIFT). Lohi et al. (2005) [22] reported that glycogen synthase can be inactivated through GSK3 by laforin (EPM2A). The suggested pathway is polyglucosan-laforin-malin-GYS1, which removes GYS1 via proteasomal degradation. The variant is in a loop far from the active site, so it is unlikely that it could exert an inhibitory effect on GYS1 and impact glycogen synthesis.

The S variants observed in genes related to glycogen synthesis, in single cases or with an incidence different from the general population (Table 5a), do not correlate with the z-score for any of the clinical parameters tested, as expected for variants not impacting the protein structure (unless the sequence variation impacts splicing). In patients carrying NS variants, the z-score for ws is at or below the mean of the whole group, and the z score for 6 mwt and FVC is at or above the mean in four out of six cases, but, in addition to the differences already discussed in the results for ws vs. 6 mwt/FVC, no other clear correlation can be observed between the available clinical data and any of the variants identified.

The z-score for 6 mwt and FVC generally behaved similarly, being positive or negative without major quantitative differences. The differences between ws vs. 6 mwt/FVC for cases FM63, SM04, (S variants) and BR04, GG01 (NS variants) (Table 5a), reproduce the differences already mentioned above for specific mutations (Table 2) and suggest that a parameter that gives a global representation of the phenotype, such as ws, is of little use in describing the phenotype–genotype correlation.

In the set of genes related to glycogen synthesis, S and NS variants observed in single cases or with an incidence different from that of the general population are equally represented (Table 4a: S, n = five in six cases and NS, n = four in seven cases). The rs117639846 NS variant, was defined as benign/tolerated in gnomAD, and the four cases carrying it (BA51, BR01, US01, and GG01, Table 4a) showed both mild and severe phenotypes (Table 1).

In the set of genes related to glycogen catabolism, most of the variants observed in the single cases or with an incidence different from the general population were NS (Table 5a: S, n = one, case BGA01_II and NS, n = six in six cases), and they were interpreted as deleterious or damaging in most cases.

Among the eight cases with a ws 1 SD above the mean (Table 1), CA01 carries a NS variant rs370391954 in AGL, defined as deleterious or probably damaging in gnomAD; the patient also shows poor performances in 6 mwt and FVC (Table 1 and Table 2).

Case SM01 was among the six cases whereby 6 mwt was 1 SD below the mean of the whole group and it carried a rare NS variant, rs199821084 in *GBE1*, which was interpreted as damaging in polyphen but not in SIFT; VG01 was among the six cases with FVC below a 1 SD of the mean and carried a deleterious NS variant in AGL, rs2230307 (Table 4 and Table 5).

*GYG2* is localized in Xp22.3, and twenty-eight out of thirty cases carry two or three NS variants (Table 4b), two of which are consistently defined as benign (rs2306734 and rs2306735). No sex differences in the clinical presentation seem to be related to any of the SNVs for this gene; in fact, the two cases not carrying any SNV (CA01 and VG01) showed poor clinical outcomes (Table 1), while the male patients in whom an additional variant was referred by SIFT as deleterious (rs11797037) showed a mild presentation.

Interestingly, in the genes related to the first steps of glycogen synthesis that were active in muscle (*UGP2, GYG1*, and *GYS1*), LOPD patients carried no or only synonymous variants; whereas, in genes with a similar biochemical activity that were was active in the liver, (*GYG2* and *GYS2*), the NS variants are largely present. The lack of NS variants in *GYG1* versus *GYG2* does not seem to be related to the general distribution of variants in the genes, as the z-scores for missense variants are very similar (Table 3a). *GYS1* had a high z-score, indicating that it was less able to bear a significant number of the NS variants (Table 3a).

The analysis of genes related to the control of glycogen synthesis showed that, in keeping with a high Z score for NS variants (Table 3a), *GSK3A* presented only S variants; two cases, (BGA01_II and BGA02) carry, in addition to a common variant, also a rare synonymous variant (rs377139858), but without a homogeneous clinical presentation.

Only two cases, BR04 and FM63 (Table 4a), carried one NS and one S variant in GSK3B, respectively, and BR04 was among the cases with no clinical evidence of the disease.

In genes related to glycogen catabolism, *PYGB, PYGL*, and *PYGM*, only *PYGM* is expressed in the muscle, and when carrying biallelic mutations it causes McArdle disease (glycogen storage disease type V).

All of them share very similar z-scores for the NS variants (Table 3b), indicating that almost all the possible variants are present in the population, and in fact, the NS variants are present in a consistent number of cases for *PYGB* and *PYGL* (Table 3b).

Conversely, only one out of thirty cases (MN02, a female) carried an NS variant for *PYGM*, and this patient showed poor respiratory performance and needed respiratory support at night (Table 1). The variant is reported as deleterious (SIFT) or possibly damaging (polyphen), but it is of an uncertain significance in other databases. At least in theory, if in fact this heterozygous variant has even a slight impact in terms of reducing glycogen catabolism outside lysosomes, it might add to the phenotype of the patient, in keeping with our working hypothesis. Of course, the observed major burden on the respiratory function compared to muscular function remains to be explained, and a larger number of cases need to be studied.

The apparently smaller number of NS variants in genes of glycogen synthesis specifically expressed in muscle vs. genes with the same biochemical function, but expressed in other tissues (*UGP2, GYG1*, and *GYS1* vs. *GYG2* and *GYS2*), deserves further biochemical work at the cellular level to fully understand its significance.

A possible hypothesis is that in PD, a phenotype such as LOPD is more likely to be observed in the absence of NS variants in the genes involved in glycogen metabolism.

## 5. Conclusions

To the best of our knowledge, this is the only series of cases of LOPD in which the variants in genes related to glycogen synthesis and catabolism were investigated.

Our data confirm that even in a group of patients bearing the same mutation on allele #1, the [c.-32-13T>G], which allows for the production of a small amount of normally spliced proteins (Dardis et al., 2014) [23], the type of mutation present in allele #2 is not sufficient to provide a clear genotype–phenotype correlation.

This observation is well in keeping with the data reported by Musumeci et al. [7], who reported large clinical variation (onset age 12 to 55 years) and course even in cases with a homozygous [c.-32-13T>G] mutation; in addition, they calculated that the mutation is observed more rarely than expected and interpreted this phenomenon as being caused by reduced penetrance.

In our exploratory analysis of exonic variants in genes related to glycogen synthesis and catabolism, we observed a reduced number of variants in the genes expressed in muscles compared to genes expressed in other tissues. As expected, we did not find a single variant strongly affecting the phenotype, and we believe that, after more extensive work on a set of genes relevant for general cellular function, such as autophagy, or pathways of muscle structure, and repair and respiratory function, it will be possible to identify a “genetic signature” that is relevant to explain, at least in part, the genetic variability, including the course of the disease and response to ERT.

It is of utmost Importance to enrich the details of the clinical problems in these patients as well as to find a score that correctly expresses the disease progression or disease pattern. For instance, any motor/respiratory quantitative scores certainly provide data that are relevant for diagnosis, follow-up, and evaluation of the success of ERT; however, they are probably not enough to describe muscle impairment in such details as to make a correlation with genes, whose even slightly reduced function, due to genetic variants, has an impact on the phenotype.

A measure taking into account both the severity of any (motor/respiratory) clinical score and the patients’ disease duration/actual age, could better reflect the phenotype in “dynamic” terms of disease progression and pattern, at least ideally.

Newborn screenings for a number of different diseases are becoming more common in recent years, and the largest screening for Pompe disease has been recently reported [24]. The authors have extensively addressed the problem of newborns carrying mutations that are associated with late-onset disease, and have provided their suggestions for the clinical follow-up of these cases. Studies that aim to identify modifier genes and to better define the clinical parameters to be assessed during follow-up will be instrumental in dealing with the new challenges (counselling, treatment, and type and timing of clinical work-up) that are proposed by the very early, preclinical identification of biallelic mutations in Pompe disease.

In the long term, improving our knowledge of genetic variability and its relationship to phenotypic variability may be useful for the development of new therapeutic approaches.

## Figures and Tables

**Table 1:** Summary of demographic, genetic, and clinical data. Allele 1: IVS1 = c.32-13T>G; WS = Walton score; 6 mwt: 6 min walking test; FVC: forced vital capacity; walking support: 0 = none; 1 = cane; 2 = walker; respiratory support: 0 = none; 1 = NIV (noninvasive ventilation) at night; 2 = NIV day and night. #: not measured because of severe coxarthrosis.

	Sex	Age of Onset	Age at Diagnosis	Allele 1	Allele 2	WS	6 mwt (%)	FVC (%)	Walking Support	Ventilatory Support
BA51	M	49	63	IVS1	c.1927G>A	3	23.00	35.00	0	2
BGA01_II	F	25	23	IVS1	c.525delT	3	69.00	94.00	0	0
BGA02	F	7–10	37	IVS1	c.525delT	5–6	52.00	77.00	1	0
BR01	F	39	41	IVS1	c.2237G>A	3	75.00	105.00	0	0
BR04	M	71	43	IVS1	c.2237G>A	0	100.00	100.00	0	0
CA01	M	35	46	IVS1	c.2237G>A	5	53.00	43.00	0	1
DA01	M	27	29	IVS1	c.1121G>T	2	99.00	77.00	0	0
DD45	M	30	66	IVS1	c.1927G>A	3	58.00	54.00	0	0
DR01	F	20	28	IVS1	c.1802C>G	3	49.00	53.00	1	1
FB01	M	44	45	IVS1	c.2237G>A	6	1.00	42.00	1	1
FM63	F	< 25	36	IVS1	c.1564C>G	5	21.00	55.00	1	1
FR01	M	26	28	IVS1	c.2237G>A	1	61.00	86.00	0	0
GAA81	F	13	13	IVS1	c.2237G>A	6	26.00	53.00	1	1
GG01	F	26	37	IVS1	c.784G>A	1	96.00	100.00	0	0
GS01	F	15	15	IVS1	c.784G>A	5	38.00	67.00	0	0
IM01	M	65	71	IVS1	c.525delT	2	103.00	86.00	1	1
ME01	F	30	35	IVS1	c.1465G>A	6	8.00	82.00	2	0
MN01	M	43	45	IVS1	c.1551 + 1G>C	2	79.00	85.00	0	0
MN02	F	38	54	IVS1	c.1551 + 1G>C	2	84.00	55.00	0	1
MN03	F	25	52	IVS1	c.1551 + 1G>C	2	56.00	46.00	0	1
PT01	F	37	43	IVS1	c.1465G>A	3	47.00	45.00	0	1
RA01	F	38	39	IVS1	c.2481 + 102_2646 + 31del	5	57.00	81.00	1	0
SA01	F	35	50	IVS1	c.784G>A	3	50.00	72.00	1	0
SM01	F	45	76	IVS1	c.784G>A	3	30.00	47.00	0	1
SM02	M	71	61	IVS1	c.2237G>A	0	100.00	100.00	0	0
SM04	M	71	51	IVS1	c.2237G>A	0	100.00	100.00	0	0
TL55	M	25	57	IVS1	c.1655T>C	#	#	65.00	#	0
US01	F	40	51	IVS1	c.525delT	3	78.00	97.00	0	1
VG01	M	10	31	IVS1	c.1927G>A	3	60.00	37.00	0	1
ZA01	F	15	15	IVS1	c.1927G>A	0	110.00	91.00	0	0

**Table 2:** Clinical data in sub groups of patients carrying different mutations on the second allele.

Mutation (n)	Walton Score	6 mwt (%)	FVC (%)	Walking Device None/Cane/Walker	Respiratory Support None/Night/Day and Night
All (30)	2.93 ± 1.85	61.17 ± 30.21	71.07 ± 22.29	21/8/1	16/13/1
Very severe (15)	2.66 ± 2.05	68.53 ± 29.04	78.07 ± 23.20	11/4/0	8/7/0
c.525delT (4)	3.25 ± 1.25	73.25 ± 22.61	88.50 ± 8.96	2/2/0	2/0/1
c.2237G>A (8)	2.62 ± 2.72	64.50 ± 36.93	78.88 ± 27.38	6/2/0	5/3/0
c.1551 + 1G>C (3)	2 ± 0	73.0 ± 14.93	62.0 ± 20.42	3/0/0	1/2/0
Potentially less severe (15)	3.21 ± 1.62	53.28 ± 31.31	64.07 ± 19.64	10/4/1	9/5/1
Potentially less severe (-other) (10)	3.00 ± 1.70	52.00 ± 31.42	63.00 ± 22.92		
c.1927G>A (4)	2.25 ± 1.5	62.75 ± 35.78	54.25 ± 25.94	4/0/0	2/1/1
c.784G>A (4)	3 ± 1.63	53.5 ± 29.50	71.5 ± 21.86	3/1/2	3/1/0
c.1465G>A (2)	4.5	27.5	45	1/0/1	1/1/0
Other (5)	3.75 ± 1.50	56.5 ± 32.26	66.25 ± 12.61	3/1/0	3/1/0

**Table 3:** a and b: Expected vs. observed (e/o) variants and Z score for synonymous (S) and non-synonymous (NS) variants for genes related to glycogen synthesis and catabolism (from gnomAD), and number of cases with no, synonymous, or non-synonymous variants. When the total number of variants exceeds 30, this implies that some cases carry more than one type of variant (see Table 4 and Table 5).

3a-Synthesis	S	NS	None	S	NS
Genes					
*UGP2*	97/105 (z = −0.6)	269/180 (z = 1.93)	29	1	--
*GYG1*-m	75.8/68 (z = 0.7)	187.5/185 (z 0.06)	16	14	--
*GYG2*-l	95.7/96 (z −0.02)	202/199 (z 0.08)	2	--	28
*GYS1*-m	199.6/183 (z 0.92)	472.5/360 (z 1.84)	23	7	--
*GYS2*-l	139.2/166 (z −1.79)	381.6/374 (z 0.14)	2	--	28
*GBE1*	127.4/117 (z 0.73)	356.9/352 (z 0.09)	--	1	30
*EPM2A*	61.6/78 (z 1.64)	155.9/165 (z −026)	20	10	--
*NHLRC1*	107.5/112 (z −0.34)	240.6/219 (z 0.5)	9	7	16
*GSK3A*	103.5/90 (z 1.04)	244.6/103 (z 3.22)	--	30	--
*GSK3B*	87.2/80 (z 0.61)	238.6/117 (z 2.8)	28	1	1
3b-catabolism	S	NS	none	S	NS
Genes					
*PYGB*	242.1/272 (z −1.51)	551.9/555 (z −0.05)	13	17	13
*PYGL*	182.1/177 (z 0.3)	465.7/478 (z −0.2)	16	14	9
*PYGM*	211.4/221 (z −0.52)	522/519 (z 0.05)	28	1	1
*AGL*	263.9/268 (z −0.2)	793.4/830 (z −0.46)	1	28	12

**Table 4:** **a**: Exonic variants found in genes acting on glycogen synthesis. The incidence in the population (gnomAD) and in the sample studied was compared using Fisher’s exact test for count data due to the small sample size of observations for many of the alleles analyzed; differences were considered to be significant (*) after Bonferroni correction, with *p*-value equal or below 0.0013 ⋍ 0.05/37. The codes of the single cases carrying the variant or showing significant differences in incidence vs. the general population are in bold. Syn: synonymous; NSyn: non-synonymous. For NSyn variant the clinical significance (SIFT: d = deleterious or t = tolerated; polyphen: pr = probably damaging; po = possibly damaging. or b = benign; na = not available) is also entered. **b**: Exonic variants found in genes acting on glycogen catabolism. Incidence in the population (gnomAD) and in the sample studied. Fisher’s exact test for count data was used due to the small sample size of observations for many of the alleles analyzed; differences were considered to be significant after Bonferroni correction, with *p*-value equal or below 0.0013 ⋍ 0.05/37. The codes codes of the single cases carrying the variant or showing significant differences in incidence vs. the general population are in bold. For NSyn variant the clinical significance (SIFT: d = deleterious or t = tolerated; polyphen: pr = probably damaging; po = possibly damaging. Or b = benign; na = not available) is also entered.

**a**						
**Gene**	**Type of Variant**	**Variant Code**	**General Population** **Allele Frequency %** **(Allele Count/Number)**	**Patients** **Allele Frequency %** **(Allele Count/Number)**	***p*-Value**	
*UGP2*	Syn	rs752208825 2-64114565-A-G	0.0000239 (6/251092)	0.01 (1/60) VG01	0.00167	
*GYG1*	Syn	rs4938 3-148727133-G-A	0.3131 (88538/282742)	0.23 (14/60)	0.211	
	Syn	rs148619511 3-148714104-A-G	0.00717 (2028/282634)	0.01 (1/60) SM04	0.3509	
*GYG2*	NSyn	rs2306734 X-2777985-C-T	0.6761 (133803/197917)	(0.38) 23/60	0.00001 *	b/t
	NSyn	rs2306735 X-2779570-A-G	0.6584 (2132253/200876)	(0.38) 23/60	1.556 × 10^−5^ *	b/t
	NSyn	rs11797037 X-2761050-C-T	0.1506 (16000/106230)	(0.06) 4/60	0.07129	d/b
	Syn	rs145525914 X-2793922-G-C	0.0001929 (35/181460)	(0.01) 1/60 GG01	0.01183	
*GYS1*	Syn	rs5464 19-49485548-G-A	0.2713 (76723/282804)	(0.11) 7/60	0.005437	
*GYS2*	NSyn	rs117639846 12-21690035-C-G	0.02095 (5921/282568)	(0.06) 4/60 BA51; BR01; US01; GG01	0.0372	b/t
	NSyn	rs2306180 12-21713402-T-C	0.7680 (217081/282672)	(0.45) 27/60	1.072 × 10^−7^ *	b/t
*GBE1*	NSyn	rs2172397 3-81643167-T-C	0.9752 (208612/213910)	(0.50) 30/60	2.2 × 10^−16^ *	b/t
	NSyn	rs2229519 3-81698130-T-C	0.3185 (80786/253676)	(0.18) 11/60	0.0259	d/b
	Syn	rs2229520 3-81586149-G-A	0.004988 (1307/262054)	(0.01) 1/60 MN03	0.2593	
	NSyn	rs1998210 3-81640256-A-T	0.00009506 (26/273500)	(0.01) 1/60 SM01	0.00590	t/pr
*NHLRC1*	NSyn	rs10949483 6-18122506-G-A	0.3980 (101008/253800)	0.26 (16/60)	0.04695	d/b
	Syn	rs115931931 6-18122526-A-G	0.08902 (21631/242992)	0.11 (7/60)	0.4914	
*GSK3A*	Syn	rs851609 19-42736267-A-G	0.9996 (280773/280880)	0.5 (30/60)	2.2 × 10^−16^ *	
	Syn	rs377139858 19-42746342-C-T	0.0002832 (27/95338)	0.03 (2/60) BGA01_II; BGA02	0.000015 *	
*GSK3B*	NSyn	rs34009575 3-119585455-A-T	0.0001439 (40/277952)	0.01 (1/60) BR04	0.008811	t/b
	Syn	rs72546694 3-119634947-C-T	0.0006164 (174/282284)	0.01 (1/60) FM63	0.0365	
*EPM2A*	Syn	rs35230590 6-146007332-C-T	0.2103 (59459/282694)	0.16 (10/60)	0.5258	
**b**						
**Gene**	**Type of Variant**	**Code**	**General Population** **Allele Frequency %** **(Allele Count/Number)**	**Patients** **Allele Frequency %** **(Allele Count/Number)**	***p***	
*PYGB*	Nsyn	rs2228976 20-25259006-G-T	0.1691 (47785/282596)	0.21 (13/60)	0.3042	d/pr
	Syn	rs2227890 20-25260931-A-G	0.4587 (129606/282540)	0.26 (16/60)	0.002718	
	Nsyn	rs201063710	not found	0.01 (1/60) BGA01_II		na
	Nsyn	rs2227891 20-25262769-G-A	0.1692 (47648/281672)	0.21 (13/60)	0.3044	t/b
	Syn	rs2227892 20-25264814-T-C	0.4586 (129539/282450)	0.26 (16/60)	0.002721	
	Syn	rs2227894 20-25255338-C-T	0.07043 (19812/281292)	0.01 (1/60)	0.128	
*PYGL*	Syn	rs15669 14-51376774-G-A	0.1555 (43969/282812)	0.13 (8/60)	0.8581	
	Nsyn	rs946616 14-51387782-C-T	0.07202 (20361/282710)	0.13 8/60 (0.13)	0.0765	t/b
	Syn	rs2075643 14-51383432-G-A	0.2311 (65366/282790)	6/60 (0.1)	0.0137	
*PYGM*	Syn	rs116812032 11-64519472-G-A	0.0003076 (87/282872)	1/60 (0.01) BGA01_II	0.01849	
	Nsyn	rs200481790 11-64519937-G-A	0.00005660 (16/282700)	1/60 (0.01) MN02	0.003601	d/po
*AGL*	Syn	rs2230306 1-100336361-C-T	0.7210 (202563/280952)	0.46 (28/60)	3.598 × 10^−5^ *	
	Nsyn	rs370391954 1-100336027-C-G	0.00003183 (8/251328)	0.01 (1/60) CA01	0.002146	d/pr
	Nsyn	rs3753494 1-100358103-C-T	0.1347 (38045/282500)	0.16 (10/60)	0.449	d/b
	Nsyn	rs141043166 1-100343254-G-A	0.008562 (2420/282634)	0.01 (1/60) TL55	0.4032	d/pr
	Nsyn	rs139488862 1-100346211-C-T	0.0005203 (147/282554)	0.03 (2/60) TL55; US01	0.000479 *	d/pr
	Nsyn	rs2230307 1-100361925-G-A	0.07178 (20297/282780)	0.01 (1/60) VG01	0.1287	d/pr

**Table 5:** **a**. Genes related to glycogen synthesis: Z score for Walton score, 6 mwt, and FVC for cases whereby a single variant is present for a specific gene or the variant has a significant difference in incidence vs. the general population. S = synonymous; NS = non-synonymous. **b**. Genes related to glycogen catabolism: Z score for Walton score, 6 mwt, and FVC f cases whereby a single variant is present for a specific gene or the variant has a significant difference in incidence vs. the general population. S = synonymous; NS = non-synonymous.

**a**						
**Code**	**Gene**	**Variant**		**Walton**	**6 mwt (%)**	**FVC (%)**
BGA01_II	*GSK3A*	rs377139858	S	0.03	0.25	1.03
BGA02	*GSK3A*	rs377139858	S	1.38	−0.29	0.26
FM63	*GSK3B*	rs72546694	S	1.15	−1.32	−0.71
MN03	*GBE1*	rs2229520	S	−0.50	−0.17	−1.12
SM04	*GYG1*	rs148619511	S	−1.58	1.28	1.30
VG01	*UGP2*	rs752208825	S	0.03	−0.03	−1.52
BA51	*GYS2*	rs117639846	NS	0.03	−1.23	−1.61
BR01	*GYS2*	rs117639846	NS	0.03	0.45	1.52
BR04	*GSK3B*	rs34009575	NS	−1.58	1.28	1.30
GG01	*GYG2*	rs145525914	NS	−1.04	1.15	1.30
	*GYS2*	rs117639846	NS	−1.04	1.15	1.30
SM01	*GBE1*	rs199821084	NS	0.03	−1.03	−1.07
US01	*GYS2*	rs117639846	NS	0.03	0.55	1.16
**b**						
**Code**	**Gene**	**Variant**		**Walton**	**6 mwt (%)**	**FVC (%)**
BGA01_II	*PGB*	rs201063710	NS	0.03	0.25	1.03
CA01	*AGL*	rs370391954	NS	1.15	−0.47	−1.25
MN02	*PYGM*	rs200481790	NS	−0.50	0.75	−0.71
TL55	*AGL*	rs141043166	NS	#	#	−0.26
	*AGL*	rs139488862	NS	#	#	−0.26
US01	*AGL*	rs139488862	NS	0.03	0.55	1.16
VG01	*AGL*	rs2230307	NS	0.03	−0.03	−1.52
BGA01_II	*PYGM*	rs116812032	S	0.03	0.25	1.03

## Data Availability

Not applicable.
